# Modeling and analyzing the action process of monoamine hormones in depression: a Petri nets-based intelligent approach

**DOI:** 10.3389/fdata.2023.1268503

**Published:** 2023-09-25

**Authors:** Xuyue Wang, Wangyang Yu, Chao Zhang, Jia Wang, Fei Hao, Jin Li, Jing Zhang

**Affiliations:** ^1^Key Laboratory of Intelligent Computing and Service Technology for Folk Song, Ministry of Culture and Tourism, Shaanxi Normal University, Xi'An, China; ^2^School of Computer Science, Shaanxi Normal University, Xi'An, China; ^3^Intelligent Policing Key Laboratory of Sichuan Province, Sichuan Police College, Luzhou, China; ^4^School of Information Construction and Management Department, Shaanxi Normal University, Xi'An, China

**Keywords:** Petri nets, intelligent computing, healthcare, visualization, data technologies

## Abstract

In contemporary society, the incidence of depression is increasing significantly around the world. At present, most of the treatment methods for depression are psychological counseling and drug therapy. However, this approach does not allow patients to visualize the logic of hormones at the pathological level. In order to better apply intelligence computing methods to the medical field, and to more easily analyze the relationship between norepinephrine and dopamine in depression, it is necessary to build an interpretable graphical model to analyze this relationship which is of great significance to help discover new treatment ideas and potential drug targets. Petri net (PN) is a mathematical and graphic tool used to simulate and study complex system processes. This article utilizes PN to study the relationship between norepinephrine and dopamine in depression. We use PN to model the relationship between the norepinephrine and dopamine, and then use the invariant method of PN to verify and analyze it. The mathematical model proposed in this article can explain the complex pathogenesis of depression and visualize the process of intracellular hormone-induced state changes. Finally, the experiment result suggests that our method provides some possible research directions and approaches for the development of antidepressant drugs.

## 1. Introduction

Major depression is a common and costly disorder. It is usually associated with severe and persistent symptoms that may eventually lead to compromised life and property safety, and even death (Hasler, 2010). The current high incidence of depression remains a concern. Due to the individual differences that exist in the etiology of major depressive disorder, the pathophysiological mechanism has been difficult to clarify. Some techniques have been applied to depression diagnosis to make it easier to analyze (Joshi and Kanoongo, 2022; Liu et al., 2023; Marwaha et al., 2023). Moreover, there are some neurobiology theories involved in the treatment of depression, and some potential treatments have been proposed in clinical applications (Gidron and Ronson, 2008; Berezantsev, 2017). These theories are based on studies of psychosocial stress, neurotransmitters, circadian rhythms, and sleep quality (Mehraei, 2017, 2018). However, the current problem is that there is no suitable model to analyze the pathological effects and internal logic of depression-related hormones. Inspired by these existing theoretical studies, we intend to do further research on the interactions of related hormones in depression. Therefore, we need a model to explain the relationship between these monoamine hormones and use some effective methods to analyze and elucidate their physiological characteristics.

Petri net (PN) (Wu, 2006) is a modeling and analysis tool for distributed systems. As a system model, PN can not only depict the structures of a system but also describe the dynamic behaviors to study complex theory (Yu et al., 2020). At present, some works have applied PN to the medical field. For example, Nadji-Tehrani and Eslami (2020) proposes a new pathway analysis method to model the signaling pathways based on PN. In Ming and Hofestadt (2006), the authors proposed a PN model to estimate genomic and regulatory metabolic levels. In Gupta et al. (2021), the bioenergetics of mycobacterium tuberculosis are studied with and without uncouplers using PN. At the same time, PNs are also widely used in clinical pathways and medical system service (Wu and Yang, 2007; Zhao et al., 2010; Yu et al., 2018a).

As of now, most of the research on diseases is dedicated to diagnostic and prognostic aspects, but also involves biochemical and cellular microscopic levels (Mor et al., 2005; Tanguay-Sela et al., 2022). In many diseases, depression is treatable using a variety of modalities that include psychotherapy, pharmacotherapy, and electroconvulsive therapy (BEC, 1998). In order to suggest some possible processes and pathways to study as targets for the development of anti-depressant drugs, we need some effective analytical methods to analyze the relationship between norepinephrine and dopamine at the biopathological level. At present, there are few research studies that have used the invariant methods in PN to analyze the action process of monoamine hormones in depression. In Formanowicz et al. (2011), the authors build a mathematical model of the human body's iron homeostasis based on PN. Ref. Sackmann et al. (2007) presents a model of the human body iron homeostasis process based on PN with the T-invariant method. In Grafahrend-Belau et al. (2008), the authors proposed a new approach for biological classification based on T-invariant and cluster analysis.

The above studies show that PN can not only be used in the field of medical information but also partially involved in bio-pathology. However, there are few related studies on modeling and analyzing depression using PN. At the same time, there is no suitable model for modeling the roles of monoamine hormones in depression. In this article, we further explored the internal relationship of depression-related hormones and provided new research ideas for developing new treatment methods. Therefore, our main work was to introduce PN to model the interaction process between norepinephrine and dopamine. At the same time, the invariant analysis technique unique to PN was used to verify the relationship between norepinephrine and dopamine. This method not only visualizes the whole process of hormone action well but also analyzes the model using invariant techniques. This model can help medical doctors understand the specific action process between the two hormones. This model can be used to verify the potential mutual connections between dopamine and norepinephrine. The main contributions of this article are as follows:

(1) We constructed a model of dopamine and norepinephrine in depression based on PN, which can visualize the structure of the entire hormone change process well, and dynamically simulate the running process and output relevant results.

(2) We used the invariant method of PN to verify a potential interlink between dopamine and norepinephrine in the effects of depression. Meanwhile, we compared the simulation results and verified the reasonableness of the model. This can help doctors to understand the specific action process between dopamine and norepinephrine and to develop new treatment ideas.

The rest of this article is organized as follows: Section 2 describes the construction process of the monoamine hormones analysis model. Section 3 describes the specific analysis methods and simulation results of the model. Section 4 summarizes the full article.

## 2. Modeling scheme

In this section, we present the relevant definitions and analytical methods, including the specific modeling process of depression, the specific structures used in constructing the depressive hormones interaction process, and the specific analysis results from the invariant analysis technique.

### 2.1. Related concepts

#### 2.1.1. Petri nets

PN is a modeling and analysis tool for distributed systems. It is particularly convenient for describing the sequence, concurrency, conflict, and synchronization of system processes (Yu et al., 2018b; Wang et al., 2022). PN can provide the graphical notations and the basic primitives for modeling concurrency, communication, and synchronization. The following are the related definitions of PN.

*Definition 1* (Wu, 2006): A *N* is a four-tuple, *N* = (*S, T; F, M*),

Where

1) *S* is a finite set of places.

2) *T* is a finite set of transitions, *T* ∩ *S* = ∅, *S* ∪ *P* ≠ ∅.

3) *F* ⊆ (*S* × *T*) ∩ (*T* × *S*) is a set of directed arcs from transitions to places and from places to transitions. Where, dom (*F*) ∪ cod (*F*) = *S* ∪ *T*: dom (*F*) = {*x* ∈ *S* ∪ *T* | ∃ *y* ∈ *S* ∪ *T* : (*x, y*) ∈ *F*}, cod (*F*) = {*x* ∈ *S* ∪ *T* | ∃ *y* ∈ *S* ∪ *T* : (*y, x*) ∈ *F*}.

4) *M*: *S* → { 0, 1, 2, ⋯  } is the marking of *N*.

*Definition 2* (Wu, 2006): The following are the transition firing rules of PN.

1) For transition *t* ∈ *T*, if ∀ *s* ∈ *S* : *s* ∈ ^•^ *t* → *M*(*s*) ≥ 1, which explains the transition t is enabled in the marked *M*, denoted as *M*[*t* >.

2) If transition *t* is enabled, it may be fired; and it will generate a new marking *M*′, denoted as [*t* > *M*′.

For ∀ *s* ∈ *S*:


(1) $$M^{\prime }(s)=\{\begin{array}{l} M(s)-1,s\in^{•}t-t^{•}\\ M(s)+1,s\in t^{•}{-}^{•}t\\ M(s),\;other\;cases \end{array}$$

#### 2.1.2. Incidence matrix

The main advantage of using PN for modeling real systems is that the properties of the model system can be analyzed by the unique analysis techniques of PN. Typically, system models based on PN have intuitive graphical representations and rigorous formal processes. If a PN model exactly describes the structures and operations of a system, then some properties of the system will also be reflected in its PN model. To better dissect the system structures and behaviors, PN also provides some analysis methods, mainly including reachable marking graphs, coverability trees, incidence matrices, and state equations. When the PN can be represented by a matrix, the method of linear algebra can be introduced to analyze the properties of the PN. Meanwhile, the incidence matrix of PN corresponds to the matrix of hormonal action behavior in a metabolic network. The incidence matrix comprises the change in token amount for each place when a single transition of the whole network fires.

*Definition 3* (Wu, 2006): If *N* = (*S, T*; *F, M*) is a PN, *S*={*s*_1_, *s*_2_, ⋯ , *s*_*m*_}, *T*={*t*_1_, *t*_2_, *t*_3_, ⋯ , *t*_*n*_}, then *N* can be represented as a incidence matrix $A=A^{+}-A^{-}={\left[a_{ij}\right]}_{n\times m}$, where *A*^+^ and *A*^−^ are called the output matrix and input matrix of *N*:


(2) $$\begin{array}{l} a_{ij}=a_{ij}^{+}-a_{ij}^{-},i\in \left\{1,2,\cdots \;,n\right\},j\in \left\{1,2,\cdots \;,m\right\} \end{array}$$

(3) $$A^{+}=a_{ij}^{+}=\{\begin{array}{l} 1,\;if\;(t_{i},s_{j})\in F\\ 0,\;else \end{array}$$

(4) $$A^{-}=a_{ij}^{-}=\{\begin{array}{l} 1,\;if\;(s_{j},t_{i})\in F\\ 0,\;else \end{array}$$

#### 2.1.3. Invariant

PNs also have some structural properties, which are determined by the structures and have nothing to do with the initial marking. The structural properties mainly include liveness monotonicity, S-invariant and T-invariant, repeatable vector, deadlock, and trap. Since such properties are determined by the structure of the PN, most of them can be determined by the incidence matrix, and a relatively complete analysis result can be obtained. The following is the definition of the invariants.

*Definition 4* (Wu, 2006): If *N* = (*S, T*; *F*) is a PN, *S*={*s*_1_, *s*_2_, ⋯ , *s*_*m*_}, *T*={*t*_1_, *t*_2_, *t*_3_, ⋯ , *t*_*n*_}, *A* is the incidence matrix of *N*, then:

1) If ∃ *Y* = (*y*_1_, *y*_2_, *y*_3_, ⋯ , *y*_*m*_) > 0, *m* ∈ **N**^+^, *AY* = 0, then *Y* is the S-invariant of *N*.

2) If ∃ *X* = (*x*_1_, *x*_2_, *x*_3_, ⋯ , *x*_*n*_) > 0, *n* ∈ **N**^+^, *A*^*T*^*X* = 0, then *X* is the T-invariant of *N*.

The S-invariant represents the set of places in which the token sum remains constant throughout the execution of a PN. It represents some cyclic behaviors in metabolic pathways. The T-invariant represents the set of transitions that describe the system behavior of the network, e.g., for metabolic networks in the steady state. In this article, we will assign specific biological meanings to each transition and place.

In summary, PN not only enables visual system modeling but also can introduce many mathematical methods. For complex systems, PN can abstract each model component into places and transitions, and use formal methods to analyze the system. Furthermore, PNs have corresponding judgment methods, most of which can be converted into integer solutions of linear equations or linear inequalities to illustrate some problems. In this article, we use it to illustrate the relationship between norepinephrine and dopamine.

### 2.2. The specific modeling process

**Figure 1:**
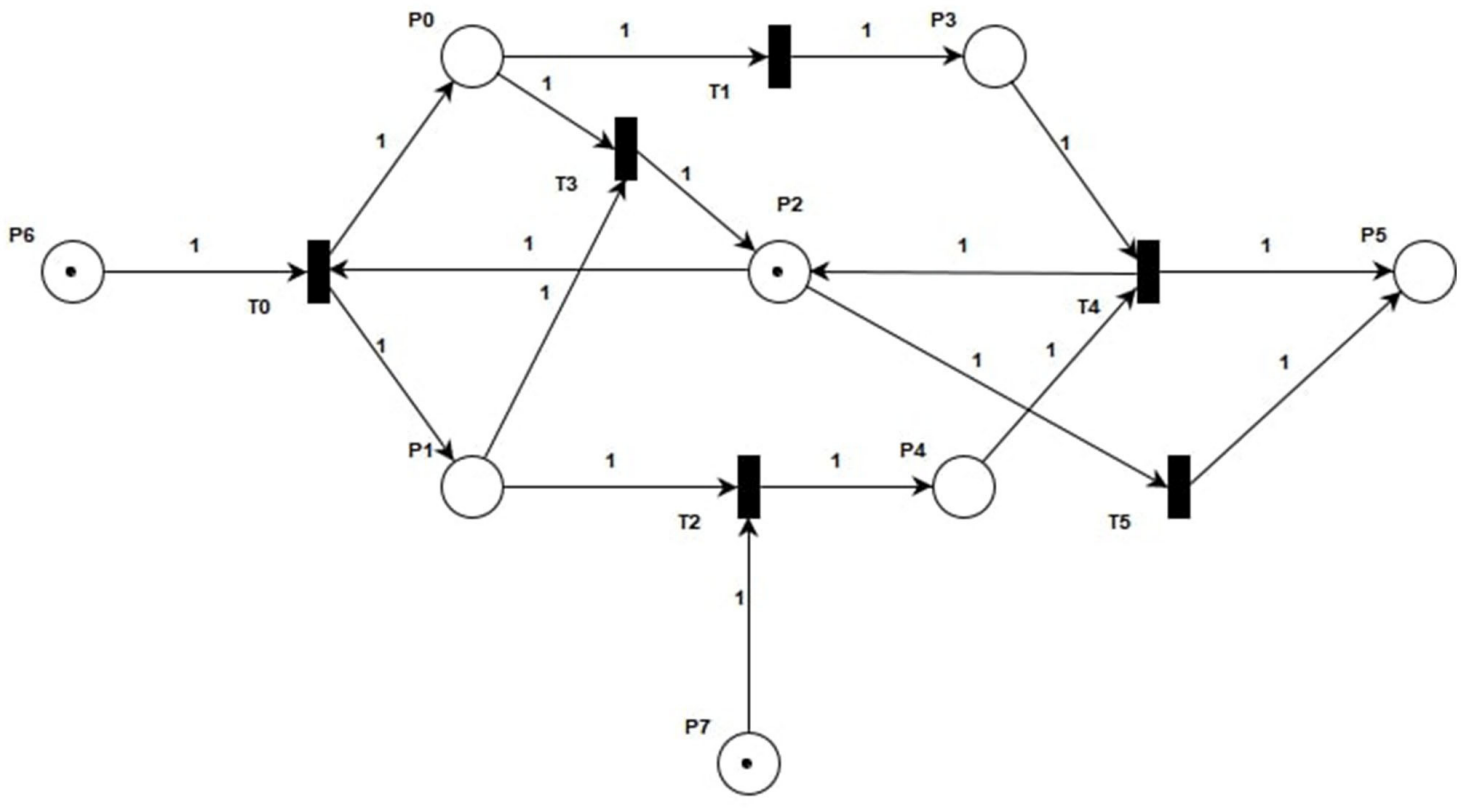
DANA model.

With the increasing suicide rate among people with depression ANG (2002), more and more scholars are paying attention to this patient group. In Section 1, we have introduced the current related research. In order to better apply PN to analyze depression, we chose to analyze the relationship between dopamine and norepinephrine in depression and model the related states. In Section 2, we have introduced the relevant definitions of PN in detail. In this section, we introduce the modeling process of a specific depression model. We constructed the *Dopamine And Norepinephrine Analyze* (DANA) model based on PN, as shown in Figure 1. In the analysis process, we will integrate the invariant technology of PN to analyze and verify the DANA model.

In Figure 1, the DANA model describes a series of state changes triggered by lower levels of norepinephrine and dopamine. There are three main types of state change. The first type is the single factor state change when dopamine is low. The second type is the single factor state change when norepinephrine is low. The third type is a multifactorial state of change when the levels of both hormones are low. The specific model structure is shown in Figure 1, with the specific meanings of the symbols shown in Tables 1, 2.

**Table 1:** The meaning of places.

**Place**	**Mean**
*P*_0_	Weakened Energy
*P*_1_	Cognitive Decline
*P*_2_	Bradykinesia
*P*_3_	intellectual disability
*P*_4_	Anhedonia
*P*_5_	Blocking Depression
*P*_6_	Norepinephrine
*P*_7_	Dopamine

**Table 2:** The meaning of transitions.

**Place**	**Mean**
*T*_0_	Low Norepinephrine Levels
*T*_1_	Decreased Willpower
*T*_2_	Low Dopamine Levels
*T*_3_	Psychomotor Block
*T*_4_	Feeling Down
*T*_5_	Thinking Slow

The basic control structure of the DANA model mainly generates subsequent states through the corresponding decision rules. As shown in Figure 1, the sequential structure describes the hormonal changes and the successive changes of several body functions. When there exists selective routing in the hormonal change process, it would be described by the concurrent structures. In this work, the control structures mean the places and transitions that are used to coordinate changes in hormone status. The internal control structures are used to construct the internal interaction process of several hormones based on the concurrent structures. The flowing tokens in the control structures are used to describe the resources needed for the action of the hormones and also describe the dynamic flow of resources in the whole process of hormone change. We can clearly see that after triggering *T*_0_ under the initial marking, new marking *M*_0_ will be generated. The arcs are used to connect associated places and transitions that belong to internal control structures, e.g., the path from *P*_6_ to *T*_0_, and *T*_0_ to *P*_0_. This denotes the internal flow of hormones. In the end, since we have given new biological meanings to each transition and library, the hormonal interaction process is further simulated by analytical techniques.

The reduction of norepinephrine leads to the insufficiency of the α_1_ receptor, which is responsible for arousal, so its insufficiency leads to decreased energy and psychomotor block, which is manifested as intellectual disability, slow movement, and decreased willpower. At the same time, it also leads to insufficiency of α_2_ receptors, which are responsible for cognition, including psychomotor operations, that is, lack of motivation and inactivity, also manifested as slow thinking. Furthermore, it also leads to insufficiency of β receptor function, which is mainly antidepressant. This leads to emotional depression. The symptoms caused by the above three receptor hypofunctions can be summarized as decreased willpower, slow thinking, and emotional depression, which together constitute the three-low depression, that is, blocking depression.

Dopamine is mainly located in three parts of the brain, the prefrontal cortex, nucleus accumbens, and striatum. *D*_1_ receptors in the prefrontal cortex govern cognition, and slow thinking results when they are deficient. The *D*_1_ receptors in the nucleus accumbens are responsible for pleasure. The pleasure associated with this receptor is anticipatory pleasure. There is also another kind of pleasure in people, relaxation pleasure. This is caused by exciting morphine receptors. Insufficient activation of receptors in the nucleus accumbens can lead to depression. The *D*_2_ receptors in the striatum are responsible for psychomotor activity, which can be understood as brain power and mobility. Their absence may lead to a decrease in willpower, that is, the brain does not function well, mobility is reduced, and one wants to do something but has no energy to do it. A lack of dopamine also leads to blocking depression. It is not clinically possible to differentiate between dopamine insufficiency and norepinephrine insufficiency in blocking depression, but the main cause of blocking depression is norepinephrine insufficiency. Therefore, treatment for this depression is mainly supplemented by norepinephrine.

The above process is reflected in Figure 1. According to the relevant definitions of PN in Section 2, we can solve the incidence matrix and invariants of the DANA model. The specific solution process is as follows. First, we obtain the correlation matrix *A*_1_ of the DANA model according to Equations (5)-(7). *A*_1_ represents the structure characteristic of the DANA model, which is the one-to-one correspondence between the incidence matrix and the structure of the PN. This is because there is at most one arc between any transitions and any places in the DANA model, and there is no situation where $a_{ij}^{+}$ and $a_{ij}^{-}$ cancel each other out.


(5) $$\begin{array}{l} A_{1}^{-}=\left[\begin{array}{cccccccc} 0 & 0 & 1 & 0 & 0 & 0 & 1 & 0\\ 1 & 0 & 0 & 0 & 0 & 0 & 0 & 0\\ 0 & 1 & 0 & 0 & 0 & 0 & 0 & 1\\ 1 & 1 & 0 & 0 & 0 & 0 & 0 & 0\\ 0 & 0 & 0 & 1 & 1 & 0 & 0 & 0\\ 0 & 0 & 1 & 0 & 0 & 0 & 0 & 0 \end{array}\right] \end{array}$$

(6) $$\begin{array}{l} A_{1}^{+}=\left[\begin{array}{cccccccc} 1 & 1 & 0 & 0 & 0 & 0 & 0 & 0\\ 0 & 0 & 0 & 1 & 0 & 0 & 0 & 0\\ 0 & 0 & 0 & 0 & 1 & 0 & 0 & 0\\ 0 & 0 & 1 & 0 & 0 & 0 & 0 & 0\\ 0 & 0 & 1 & 0 & 0 & 1 & 0 & 0\\ 0 & 0 & 0 & 0 & 0 & 1 & 0 & 0 \end{array}\right] \end{array}$$

(7) $$\begin{array}{l} A_{1}=A_{1}^{+}-A_{1}^{-}=\left[\begin{array}{cccccccc} 1 & 1 & -1 & 0 & 0 & 0 & -1 & 0\\ -1 & 0 & 0 & 1 & 0 & 0 & 0 & 0\\ 0 & -1 & 0 & 0 & 1 & 0 & 0 & -1\\ -1 & -1 & 1 & 0 & 0 & 0 & 0 & 0\\ 0 & 0 & 1 & -1 & -1 & 1 & 0 & 0\\ 0 & 0 & -1 & 0 & 0 & 1 & 0 & 0 \end{array}\right] \end{array}$$

Then, we get the S-invariants *Y*_1_ and *Y*_2_ of the DANA model according to Equations (8) and (9). We can see that the DANA model has two S-invariants. At the same time, according to Equations (11) and (13), we do not get a non-zero solution, that is to say, there is no T-invariant in the DANA model. The existence of S-invariants indicates that the DANA model has two paths during execution, and the sum of tokens in those places on that path remains constant. Since we assign biological meanings to these places, we can get results by analyzing the S-invariants.


(8) $$\begin{array}{l} AY=0 \end{array}$$

(9) $$\begin{array}{ll} \{\begin{array}{l} \;\;1y_{0}+1y_{1}-1y_{2}\;-1y_{6}\;=0\\ \;-1y_{0}\;+1y_{3}\;=0\\ \;\;-1y_{1}\;+1y_{4}\;-1y_{7}=0\\ \;-1y_{0}-1y_{1}+1y_{2}\;=0\\ \;\;+1y_{2}-1y_{3}-1y_{4}+1y_{5}\;=0\\ \;\;-1y_{2}\;+1y_{5}\;=0 \end{array} & \;\left(9\right) \end{array}$$

(10) $$\begin{array}{l} Y_{1}={\left[1,0,1,1,1,1,0,1\right]}^{T}Y_{2}={\left[0,1,1,0,2,1,0,1\right]}^{T} \end{array}$$

(11) $$\begin{array}{l} A^{T}X=0 \end{array}$$

(12) $$\begin{array}{ll} \{\begin{array}{l} \;\;1x_{0}-1x_{1}\;-1x_{3}\;=0\\ \;\;1x_{0}\;-1x_{2}-1x_{3}\;=0\\ \;-1x_{0}\;+1x_{3}+1x_{4}\;-1x_{5}=0\\ \;\;+1x_{1}\;-1x_{4}\;=0\\ \;\;+1x_{2}\;-1x_{4}\;=0\\ \;+1x_{4}\;+1x_{5}\;=0\\ \;-1x_{0}\;=0\\ \;\;-1x_{2}\;=0 \end{array} & \;\left(12\right) \end{array}$$

(13) $$\begin{array}{l} X={\left[0,0,0,0,0,0\right]}^{T} \end{array}$$

In summary, we obtained the relevant mathematical formulas that can represent the DANA model through the above solution steps. The DANA model visualizes the hormonal action process well because the PN provides the basic primitives for the graphical representation. The goal is to model a specific system with a formal modeling method, making the model more organized and the function logic of monoamine hormones in depression clearer. PN makes the mechanism more straightforward and easy to analyze and understand. Next, we will use *pipe* (201, 2019) to simulate the model and analyze the simulation results.

## 3. Model simulation and analysis

### 3.1. Model operation rules

Previously, we built a process model of monoamine hormones in depression based on PN, but model operation also needs the support of related rules. In this section, we will introduce the corresponding operation rules of the DANA model to better understand the simulation process.

In the state of the initial marking, we first take the elements of the DANA model as the input and then run it to output a new PN state. The initial marking *M*_0_ describes the initial state of the simulated system. Under *M*_0_, there may be several transitions that can be triggered, among which (at random) a transition occurs, and a new marking *M* is obtained (different transitions occur, and the obtained new marking is generally different). Under *M*_*i*_, there may be several transitions that have the right to occur, and one of them (at random) occurs, and a new marking *M*_2_ is obtained. Continuing in this way, the successive occurrences of transitions and the constant changes of markings are the running process of the DANA model. All the possible operating conditions of the DANA model can be expressed as *N* = (*N*_0_, *M*_0_), which is determined by *N*_0_ and initial marking *M*_0_. Therefore, once we are given the *N*_0_ and the *M*_0_ of the DANA model, the DANA model can be determined.

### 3.2. Model simulation

**Figure 2:**
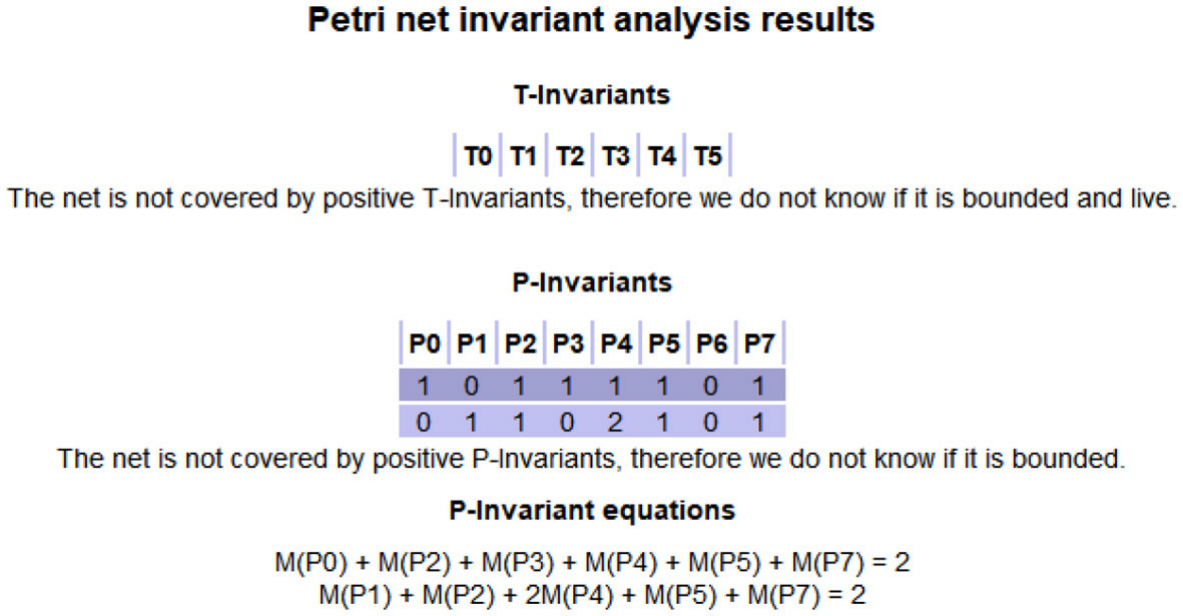
The simulation of DANA model.

To ensure the proper functionality of the developed model, we need to analyze its structure. Through the above model construction and rules analysis, we conduct model simulation by *pipe*. The simulation results of the DANA model are shown in Figure 2, which is also consistent with our solution. Fig. 2 shows that the DANA model has only two S-invariants (equivalent to the P-invariant in Figure 2) namely (1, 0, 1, 1, 1, 1, 0, 1) and (0, 1, 1, 0, 2, 1, 0, 1), and has no T-invariant.

The challenge of this work is to interpret the medical significance of each invariant and the whole model. Invariants are considered as a description of the system behaviors. It shows that the token required in this invariant is always conserved, that is, in a cycle. For the DANA model, it has two S-invariants (1, 0, 1, 1, 1, 1, 0, 1) and (0, 1, 1, 0, 2, 1, 0, 1), which respectively represent the places (*P*_0_, *P*_2_, *P*_3_, *P*_4_, *P*_5_, *P*_7_) and (*P*_1_, *P*_2_, *P*_4_, *P*_5_, *P*_7_). This means that the initial marking is placed on *P*_6_, *P*_7_, *P*_2_, and circulates only among those places (by firing *T*_0_, *T*_2_, and *T*_5_). A required number of tokens (one here) has to be put in the initial marking at a place of each S-invariant to ensure that this subnet contributes to the system behaviors. Both of its two S-invariants contain the four places *P*_2_, *P*_4_, *P*_5_, and *P*_7_. According to Tab. 1 and Tab. 2, we know the meanings of these places. This also means that both dopamine and norepinephrine can produce a state of bradykinesia, anhedonia, and blocking depression during their actions. This proves that there is an interaction and connection between the two in depression.

## 4. Discussions

In this part, we will discuss the results of model simulation. Figure 2 shows the simulation results of the DANA model based on PN. Comparing this with the results obtained in Section 2, it can be seen that the same conclusions are obtained. Under the initial marking (0, 0, 1, 0, 0, 0, 1, 1), the DANA model can be run until the end of the simulation. During this process, we found that there are several locations in the model where tokens keep recurring, which are considered invariants. The invariants mean that the model will always pass through these places during the running process.

Finally, we obtained the simulation results through modeling analysis and simulation. The results show that there is an interaction between norepinephrine and dopamine. From the whole modeling process, we can see that PN provides an intuitive graphical representation, and can transform some biological cell-level problems into mathematical ones. This is an attempt to model norepinephrine and dopamine in a formal way. It complements the artificial intelligence analysis in the field of depression research. Although our existing work still needs improvement, especially in pioneering the application of PN analysis technology in different fields, this method still provides new ideas for the study of depression treatment.

## 5. Conclusion

The main contribution of this article is to propose a new analytical method to study the interaction of dopamine and norepinephrine in depression. This method is based on the PN invariant analysis technique and reflects the behavior of the depression hormone analysis system by giving neurobiological meaning to each S-invariant. The model constructed in this article graphically shows the biological neural significance of norepinephrine and dopamine in depression. Model validation of the biochemical system was performed by evaluating and explaining some invariants. The model expands the analytical thinking of artificial intelligence in the field of depression research. The simulation result ultimately indicates some possible research processes and approaches are proposed as targets for antidepressant drug development. However, the related analysis methods are still limited and open to research. In the future, we will further explore the pathological effects of depression-related hormones and build a healthcare system with more clinical significance. In addition, more data analysis and machine learning methods should be combined with the proposed methodology in this article. The integration of data analysis or machine learning methods with the proposed approach could enhance the comprehension of the specific processes underlying the interactions between dopamine and norepinephrine.

## Data availability statement

The original contributions presented in the study are included in the article/supplementary material, further inquiries can be directed to the corresponding authors.

## Author contributions

Conceptualization and methodology: XW and WY. Validation: CZ and JW. Formal analysis: FH and JL. Data curation: JZ. All authors have read and agreed to the published version of the manuscript.
